# Microdialysis and ultrasound elastography for monitoring of localized muscular reaction after pharmacological stimulation in rats

**DOI:** 10.1186/s13104-018-3742-6

**Published:** 2018-09-03

**Authors:** Stephan Johannsen, Martin Schick, Norbert Roewer, Frank Schuster

**Affiliations:** 1 Department of Anaesthesia and Critical Care, University of Würzburg, Oberduerrbacher Str. 6, Würzburg, Germany; 2 Department of Anesthesiology and Critical Care, Medical Center-University of Freiburg, Faculty of Medicine, University of Freiburg, Hugstetter Str. 55, Freiburg, Germany

**Keywords:** Skeletal muscle, Ultrasound strain elastography, Microdialysis, Halothane, Caffeine

## Abstract

**Objective:**

Halothane and caffeine are known to cause skeletal muscular contractions in vitro and have been proven to induce circumscribed metabolic reactions when injected into rat skeletal muscle. In this study 26 rats were investigated by either continuous application of calcium 160 mM or bolus injection of caffeine 160 mM or halothane 10% vol via a microdialysis probe in the tibialis anterior muscle. Tissue elasticity at the injection site was monitored by ultrasound strain elastography. Aim of this study was to detect (I) changes in local lactate concentrations and (II) whether these can be attributed to a muscular contraction detected by ultrasound elastography.

**Results:**

Localized metabolic reactions were verified by increasing intramuscular lactate concentrations following continuous application of calcium (0.6 [0.3;0.6] to 3.6 [3.0;4.3] mmol/l after 60 min) and bolus application of caffeine (0.2 [0.2;0.3] to 1.6 [0.9;1.9] mmol/l after 30 min) and halothane (0.3 [0.1;0.3] to 4.7 [4.3;6.3] mmol/l after 30 min). However, ultrasound elastography did not detect any differences in tissue elasticity compared to control animals. The authors identified potential limitations of the study conditions, which might be crucial to avoid for future investigations.

**Electronic supplementary material:**

The online version of this article (10.1186/s13104-018-3742-6) contains supplementary material, which is available to authorized users.

## Introduction

Incubation with caffeine or halothane causes dose-dependent muscular contracture in rat skeletal muscle bundles in vitro [1, 2]. As underlying mechanism, interaction with the mammalian skeletal muscle isoform of the ryanodine receptor (RYR1), a sarcoplasmic calcium release channel, has been identified [3]. Furthermore, localized pharmacological stimulation with calcium, caffeine and halothane in rat skeletal muscle induces metabolic alterations resulting in changes of local intramuscular lactate concentrations [4]. Microdialysis technique has proven suitable to monitor regional intramuscular lactate levels following stimulation [4, 5]. Whether these metabolic changes in vivo are accompanied by localized muscular contractures is unknown so far.

Ultrasound elastography is an emerging technique for non-invasive monitoring of elastic tissue properties in real time [6]. It is well established in diagnostics of liver disease and in evaluation of breast, thyroid and prostate lesions [7–9]. In the musculoskeletal field it is increasingly used for examination of tendinopathies and soft tissue tumors [10, 11]. Changes in muscle hardness during voluntary muscular contractions can be successfully detected by ultrasound strain elastography [12].

This study investigated the muscular reaction of rat skeletal muscle to localized stimulation with calcium, halothane and caffeine. Metabolic effects were examined by microdialysis and changes in muscle hardness were simultaneously monitored by ultrasound strain elastography. The authors hypothesized that metabolic alterations of the muscle are based on localized muscular contractures which could be detected by this novel ultrasound technique in real-time.

## Main text

### Methods

#### Ethics approval

Approval was obtained from the local animal care committee (Government of Unterfranken, Würzburg, Germany, no. 63/10).

#### Anesthesia and monitoring

Anesthesia was performed by intra-peritoneal injection of ketamine (100 mg/kg) and xylazine (7 mg/kg) and repeated in 30 min intervals with one quarter of the initial dosage. Standard monitoring was established including ECG, respiratory rate, peripheral oxygen saturation and rectal temperature. An adequate arterial pulse curve obtained from peripheral pulse oximetry on the foreleg was used as surrogate parameter for sufficient hemodynamics.

#### Study procedure

**Fig. 1:**
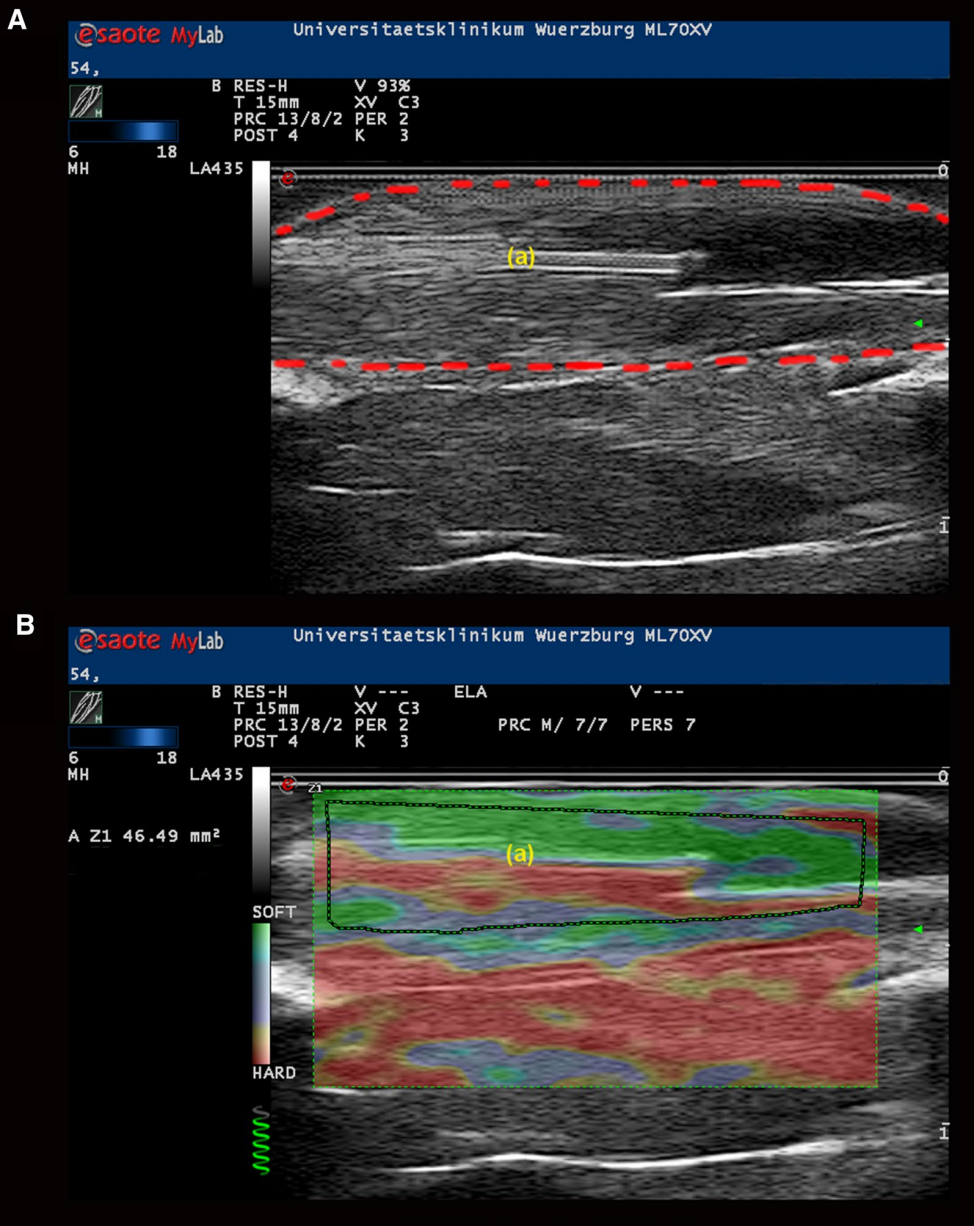
**A** B-mode ultrasound image (exemplarily) of anterior tibial muscle (red dotted line) with microdialysis probe inserted (a). **B** Superimposed color-coded strain elastography image with selected measuring field in the anterior tibial muscle (dotted line) around the microdialysis probe (a)

Both hind limbs of 26 male Sprague–Dawley rats were investigated independently. Custom build microdialysis probes with attached microtubing for targeted injection (MAB-7, Microbiotech, Stockholm, Sweden) were inserted into the tibialis anterior muscle after surgical exposure. Adequate positioning of the probes was confirmed by B-mode ultrasound (Fig. 1A). The probes were perfused with Ringer solution at 1 µl/min for a minimum equilibration period of 30 min and baseline dialysate samples were collected. For further investigation each muscle was assigned to one of seven different treatment groups (A–G). Seven muscles had to be excluded from the study due to insufficient hemodynamics of the animal (n = 6) or dysfunction of the microdialysis probe (n = 1). In group A (n = 7) perfusion was continued with Ringer solution throughout the experiment, while after equilibration in group B (n = 6) the perfusate was changed to sorbitol 160 mM and in group C (n = 6) to calcium chloride 160 mM at 1 µl/min. In groups D to G the probes were perfused with Ringer solution continuously but in addition a single 40 µl bolus of one of the following testing agents was injected into the muscle via the attached microtubing catheter: Caffeine 160 mM (group D, n = 7), Ringer solution (group E; n = 6), halothane 10% vol dissolved in soy-bean oil (group F; n = 7) and soy-bean oil only (group G; n = 6).

#### Lactate measurements

Microdialysis samples were collected in 15 min intervals. Lactate content was measured spectrophotometrically at 540 nm (Agilent 8453, Agilent Technologies, Mulgrave, Australia) after incubation of the samples with lactate reagent (Trinity biotech, Bray, Ireland) converting a chromogene dye directly correlating to the lactate concentration.

#### Ultrasound elastography

Ultrasound strain elastography is based on the analysis of tissue deformation (strain) after cyclic compression with the ultrasound transducer [13] where soft tissues exhibit higher deformation compared to stiffer areas [14]. Strain elastography was performed using a linear ultrasound transducer 6–18 MHz and the ElaXto software on the MyLab™ 70 XVision platform (Esaote S.p.A., Genoa, Italy). The transducer was attached to a custom build holder to keep it in stable position and facilitate repeated measurements. Alternating tissue deformation was induced by manual alterations of contact pressure. Light pressure followed by decompression was repeated until the feedback system of the ElaXto software indicated stable measurement (Fig. 1B). Mean elasticity was calculated by the ElaXto software and given as non-dimensional value between 0 (hard) and 100 (soft) reflecting the mean tissue elasticity of the investigated region in correlation to elasticity of the whole plane.

To evaluate whether muscular contraction in the applied setup causes alteration in muscle elasticity that could be detected by ultrasound elastography, stimulation electrodes were positioned in the tibialis anterior muscles of three different animals. Tetanic contraction was induced by an electrical stimulus (5 mA, 50 Hz) for 5 s using a nerve stimulator (Organon, Swords, Ireland). Mean elastography values were recorded at rest and during contraction. The examination was repeated once for each muscle.

For the following study the transducer was adjusted to a plane depicting the microdialysis probe centrally located within the tibialis anterior muscle (Fig. 1A). For elastographic evaluation a measuring field confined to the margins of the tibialis anterior muscle was selected (Fig. 1B). Ultrasound elastography measurements were performed before and at 1, 5, 10, 15, 20, 25, 30, 40, 50 and 60 min after starting the perfusion or after bolus application of the test substances. Differences compared to baseline elasticity were calculated for every single muscle and used for inter-individual comparison.

#### Statistical analysis

Data are presented as median and interquartile range. Normal distribution of the results was assessed by Shapiro–Wilk normality test. Comparisons were calculated between groups A to C (continuous application of test substances), between groups D and E (caffeine vs. ringer solution) and between groups F and G (halothane vs. soy-bean oil). Differences between the treatment groups were evaluated by 2-way-ANOVA followed by Sidak’s test for multiple comparisons (vital parameter, lactate, elasticity) or One-way-ANOVA (size of measuring field, weight). Paired t-test was used to compare elasticity values before and after tetanic stimulation. p < 0.05 was considered statistically significant.

### Results

The median weight of the investigated animals was 310 [295;320] g with no relevant differences between the groups. Heart rate, peripheral oxygen saturation, respiratory rate and body temperature were similar in the different groups before and after the experiment.

**Fig. 2:**
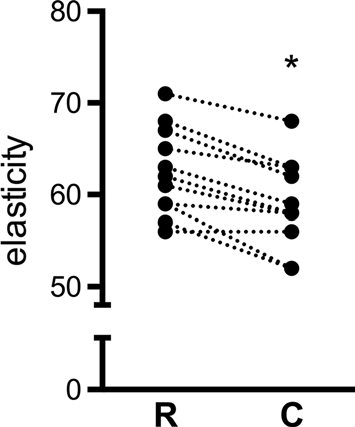
Individual mean elasticity values at rest (R) and during tetanic contraction (C) on a non-dimensional scale from 0 (hard) to 100 (soft). *p < 0.05 for differences between resting condition and contraction

Muscular contraction of the tibialis anterior muscle during tetanic stimulation was associated with significant changes in sonographically determined tissue elasticity. Median decline of elasticity values was − 4 [− 5.0;− 2.3] points during contraction compared to resting conditions (p < 0.0001) (Fig. 2, Additional file 1: Table S1).

**Fig. 3:**
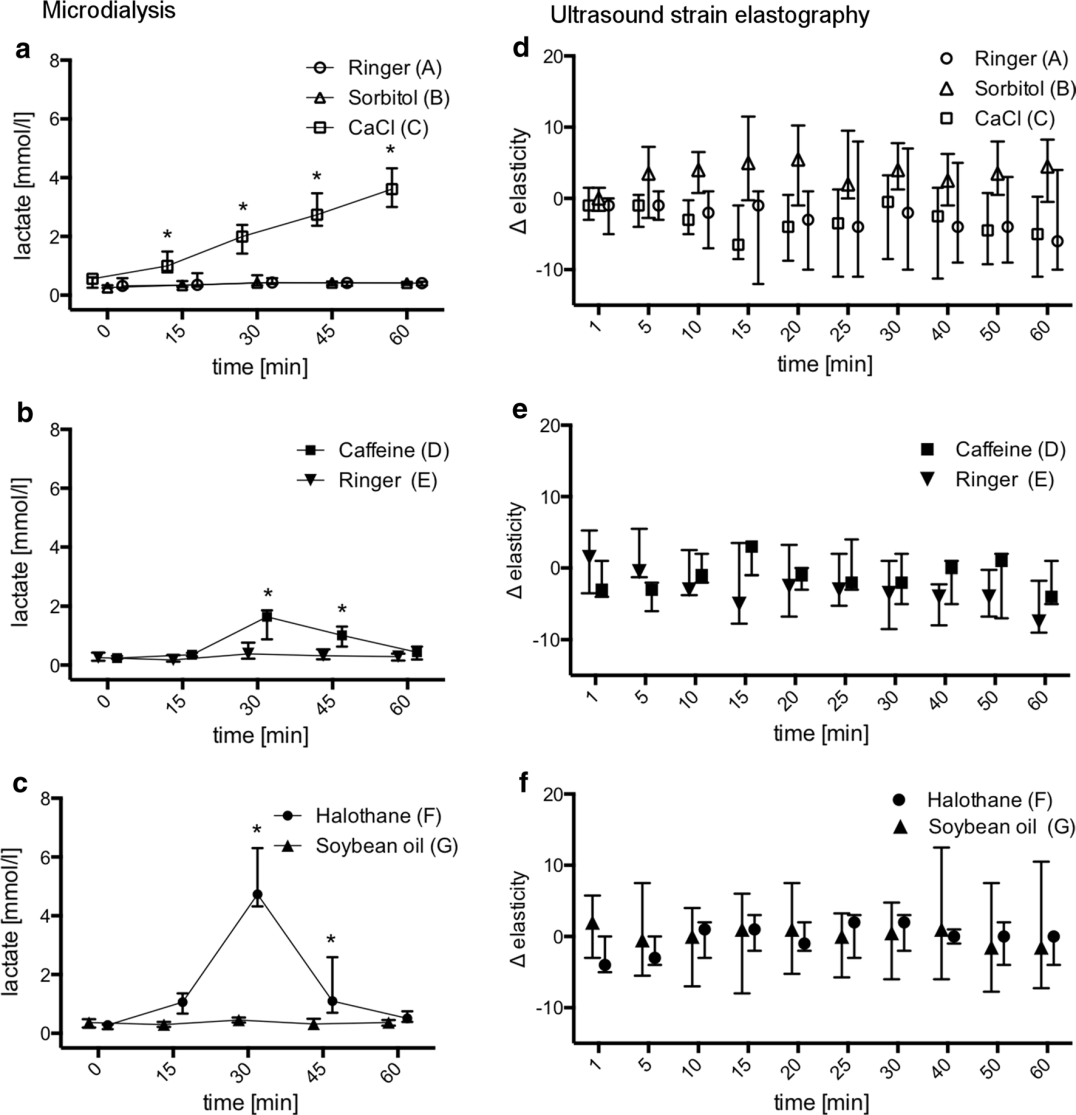
Local lactate concentrations over time measured by microdialysis after continued stimulation with Ringer solution, sorbitol 160 mM and calcium chloride 160 mM (**a**), after bolus injection of 40 µl caffeine 160 mM and Ringer solution (**b**) and after bolus injection of 40 µl halothane 10% vol dissolved in soy-bean oil and soy-bean oil only (**c**). Differences in local muscular elasticity compared to baseline measured by strain elastography on a non-dimensional scale over 60 min after pharmacological stimulation. **d** Following continued application of Ringer solution, Sorbitol 160 mM and CaCl 160 mM, **e** after bolus injection of 40 µl caffeine 160 mM and Ringer solution, **f** after bolus injection of 40 µl halothane 10 vol% dissolved in soy-bean oil and soy-bean oil only. Results presented as median and interquartile range. *p < 0.05 for differences between the investigated groups

Following application of calcium 160 mM, intramuscular lactate concentration steadily increased in group C (baseline: 0.6 [0.3;0.6], after 60 min: 3.6 [3.0;4.3] mmol/l; p < 0.0001) while it remained unaltered over the course of the experiment in group A after Ringer solution (baseline: 0.3 [0.2;0.6], after 60 min: 0.4 [0.3;0.5] mmol/l; p > 0.9999) and in group B after sorbitol 160 mM (baseline: 0.3 [0.3;0.5], after 60 min: 0.4 [0.4;0.5] mmol/l; p = 0.9935) (Fig. 3 a, Additional file 2: Table S2a). In groups D and E, lactate concentrations were significantly increased 30 and 45 min after application of caffeine 160 mM but not after Ringer solution [baseline: caffeine: 0.2 [0.2;0.3] vs. Ringer: 0.3 [0.2;0.4], at 30 min: 1.6 [0.9;1.9] (p < 0.0001) vs. 0.4 [0.2;0.8] (p = 0.8109); at 45 min: 1.0 [0.6;1.3] (p = 0.0004) vs. 0.3 [0.2;0.5] mmol/l (p = 0.9738)] (Fig. 3b, Additional file 2: Table S2b). In groups F and G, local lactate levels were again significantly increased at 30 and 45 min only in the halothane 10% vol group [baseline: halothane: 0.3 [0.1;0.3] vs. soy-bean oil: 0.4 [0.2;0.5], at 30 min: 4.7 [4.3;6.3] (p < 0.0001) vs. 0.5 [0.4;0.5] mmol/l (p = 0.9970); at 45 min: 1.1 [0.7;2.6] (p = 0.0031) vs. 0.3 [0.3;0.5] mmol/l (p > 0.9999)] (Fig. 3c, Additional file 2: Table S2c).

Sizes of the elastography measuring fields did not differ between the compared treatment groups (Groups A vs. B vs. C: 43 [35;44] vs. 39 [35;40] vs. 36 [35;41] mm^2^; Group D vs. E: 46 [42;46] vs. 42 [40;45] mm^2^ and Group F vs. G: 45 [44–48] vs. 45 [43;46] mm^2^) (Additional file 3: Table S3a–c). Mean elasticity values measured by ultrasound elastography showed only minor and inconclusive changes compared to baseline values in all investigated treatment groups A to G. In none of the groups the extent of changes after application of the testing agents was significant. There were no relevant changes in tissue elasticity determined by strain elastography after pharmacological stimulation in this setting (Fig. 3d–f; Additional file 4: Table S4a–c).

### Discussion

The presented study employed pharmacological stimulation of rat skeletal muscle with calcium, halothane and caffeine to examine localized effects in real-time. Summarizing our results, this stimulation led to (1) metabolic reactions in the form of local increase in lactate concentration measured by microdialysis while (2) ultrasound strain elastography did not detect local changes in tissue elasticity.

Pharmacological stimulation of the investigated muscle tissue by the applied testing agents proved sufficient: Application of calcium chloride 160 mM initiated calcium induced calcium liberation in skeletal muscle cells leading to hypermetabolic response and ultimately anaerobic metabolism causing significant increase in local lactate levels while Ringer solution and sorbitol 160 mM did not cause any changes. With this condition, continued influence of the trauma caused by insertion of the microdialysis probe as well as effects due to injection of a non-isotonic solution [15] could be excluded as cause for rising lactate concentration.

Bolus injection of caffeine 160 mM and halothane 10% vol triggered localized lactate increase which is attributable to muscular hypermetabolism caused by RYR1 mediated sarcoplasmic calcium release [3, 16]. Controls did not show any effects on lactate levels following Ringer solution or soy-bean oil, which excluded influence of the particular solvent or mechanical effects due to intramuscular fluid bolus application. While a previous study reported similar results in sacrificed rats that were artificially perfused with Ringer solution and showed underlying post mortem increase in systemic lactate concentrations [4], this study was performed on in vivo conditions to prevent lactate rise due to hypoxia in a dying organism.

Tetanic stimulation of the tibialis anterior muscle led to significant changes in mean muscle hardness determined by strain elastography. However, the median absolute difference compared to resting condition of 4 points on a scale from 0 to 100 was relatively small in relation to the intensity of stimulation. It is easily conceivable that smaller effect sizes could be overlooked with this technique in the investigated setup.

Strain elastography did not detect relevant differences in muscle stiffness. It remains unclear at this stage, if this technique is not sensitive enough to detect effects in this particular setup or if the metabolic effects that were proven by microdialysis were not associated with alterations in elastic tissue properties.

## Limitations

The authors identified methodological difficulties and limitations that could have affected the presented results and that should be addressed by a follow-up study.

The measuring field for elastography contained a large portion of the tibialis anterior muscle, because the exact distribution of the applied substances into the muscle was not predictable. If only a limited region of the muscle had been affected by the testing agents, calculating the mean elasticity over the whole muscle would have underestimated the effect. In contrast, tetanic stimulation affected the whole muscle and had significant impact on mean elasticity values.

Strain elastography by its nature is a qualitative rather than a quantitative technique that enables comparison of different tissue elasticity only within a particular image. For this study we evaluated images taken of the same plane at different times. Although we did not compare absolute elasticity values between investigated animals but instead used intra-individual differences over time for comparisons, the use of a quantitative elastography technique would be advantageous.

Differences in dimensions of the comparably large ultrasound transducer and the small hind limb of the rat were not ideal, although other studies using strain elastography in rats for different purposes have already been published [17, 18]. Moreover, the microdialysis probe appeared as foreign body of considerable size inside the investigated muscle. Whether these differences in dimension affected the results of the study remains unclear. However, the authors would recommend better matching of the sizes of utilized devices and investigated muscle for future studies and suggest replacing rats by a larger animal model and applying shear wave elastography instead of strain elastography to screen for quantifiable effects of localized pharmacological stimulation on muscular elasticity.

## Additional files

**Additional file 1: Table S1.** Muscle elasticity at rest and during tetanic contraction.

**Additional file 2: Table S2a.** Intramuscular lactate concentrations, groups A, B and C. **b.** Intramuscular lactate concentrations, groups D and E. **c.** Intramuscular lactate concentrations, groups F and G.

**Additional file 3: Table S3a.** Sizes of investigated areas, groups A, B and C. **b.** Sizes of investigated areas, groups D and E. **c.** Sizes of investigated areas, groups F and G.

**Additional file 4: Table S4a.** Differences in muscle elasticity, groups A, B and C. **b.** Differences in muscle elasticity, groups D and E. **c.** Differences in muscle elasticity, groups F and G.

## Abbreviations

ANOVA: analysis of variance
B-mode ultrasound: brightness modulation 2-dimensional ultrasound
mM: millimolar
RYR1: ryanodine receptor subtype 1
%vol: volume percent
