# Supplementary material for: Microdialysis and ultrasound elastography for monitoring of localized muscular reaction after pharmacological stimulation in rats
Source: BMC Res Notes. 2018 Sep 3;11:636. doi: 10.1186/s13104-018-3742-6 (PMC6122639; doi:10.1186/s13104-018-3742-6)
Supplement: Supplementary file 1 — Additional file 1: Table S1. Muscle elasticity at rest and during tetanic contraction. [file 13104_2018_3742_MOESM1_ESM.pdf]

Table S1: Muscle elasticity at rest and during tetanic contraction

	**rest**	**tetanic contraction**	p value
**muscle elasticity**	62.5 [59.0;67.8]	58.5 [56.5;63.0]	**p <0.0001**

Mean elasticity values determined by ultrasound strain elastography at rest and during tetanic contraction as non-dimensional value from 0 (hard) to 100 (soft), data presented as median and interquartile range, paired t-test for differences between resting condition and tetanic contraction.
