# Supplementary material for: Microdialysis and ultrasound elastography for monitoring of localized muscular reaction after pharmacological stimulation in rats
Source: BMC Res Notes. 2018 Sep 3;11:636. doi: 10.1186/s13104-018-3742-6 (PMC6122639; doi:10.1186/s13104-018-3742-6)
Supplement: Supplementary file 2 — Additional file 2: Table S2a. Intramuscular lactate concentrations, groups A, B and C. b. Intramuscular lactate concentrations, groups D and E. c. Intramuscular lactate concentrations, groups F and G. [file 13104_2018_3742_MOESM2_ESM.pdf]

Table S2 a: Intramuscular lactate concentrations, groups A, B and C

	**Ringer (A)**		**Sorbitol (B)**		**Calcium chloride (C)**	
**time** (min)	**lactate** (mmol/l)	p value	**lactate** (mmol/l)	p value	**lactate** (mmol/l)	p value
**0**	**0.3** [0.2;0.6]		**0.3** [0.3;0.3]		**0.6** [0.3;0.6]	
**15**	**0.4** [0.3;0.8]	>0.9999	**0.3** [0.3;0.5]	0.9998	**1.0** [0.8;1.5]	**0.0016**
**30**	**0.4** [0.4;0.6]	>0.9999	**0.4** [0.3;0.7]	0.7659	**2.0** [1.4;2.4]	**<0.0001**
**45**	**0.4** [0.3;0.5]	>0.9999	**0.4** [0.4;0.5]	0.9935	**2.7** [2.4;3.5]	**<0.0001**
**60**	**0.4** [0.3;0.5]	>0.9999	**0.4** [0.4;0.5]	0.9935	**3.6** [3.0;4.3]	**<0.0001**

Intramuscular lactate concentrations measured by microdialysis before and at different time point during continuous application of Ringer solution (A), sorbitol 160 mM (B) and calcium chloride 160 mM (D). Results presented as median and interquartile range. 2-way ANOVA with post hoc Sidak test for multiple comparisons for differences at different time points compared to baseline lactate concentration

Table S2 b: Intramuscular lactate concentrations, groups D and E

	**Caffeine (D)**		**Ringer (E)**	
**time** (min)	**lactate** (mmol/l)	p value	**lactate** (mmol/l)	p value
**0**	**0.2** [0.2;0.3]		**0.3** [0.2;0.4]	
**15**	**0.4** [0.3;0.4]	0.9396	**0.3** [0.1;0.3]	0.9962
**30**	**1.6** [0.9;1.9]	**<0.0001**	**0.4** [0.2;0.8]	0.8109
**45**	**1.0** [0.6;1.3]	**0.0004**	**0.3** [0.2;0.5]	0.9739
**60**	**0.4** [0.2;0.6]	0.6695	**0.3** [0.2;0.4]	> 0.9999

Intramuscular lactate concentrations measured by microdialysis before and at different time points following bolus application of caffeine 160 mM (D) and Ringer solution (E). Results presented as median and interquartile range. 2-way ANOVA with post hoc Sidak test for multiple comparisons for differences at different time points compared to baseline lactate concentration

Table S2 c: Intramuscular lactate concentrations, groups F and G

	**Halothane (F)**		**Soybean oil (G)**	
**time** (min)	**lactate** (mmol/l)	p value	**lactate** (mmol/l)	p value
**0**	**0.3** [0.1;0.3]		**0.4** [0.2;0.5]	
**15**	**1.1** [0.7;1.4]	0.1088	**0.3** [0.2;0.4]	>0.9999
**30**	**4.7** [4.3;6.3]	**<0.0001**	**0.5** [0.4;0.5]	0.9970
**45**	**1.1** [0.7;2.6]	**0.0031**	**0.3** [0.3;0.5]	>0.9999
**60**	**0.5** [0.4;0.8]	0.8987	**0.4** [0.3;0.5]	>0.9999

Intramuscular lactate concentrations measured by microdialysis before and at different time points following bolus application of halothane 10 %vol (F) and soybean oil (G). Results presented as median and interquartile range. 2-way ANOVA with post hoc Sidak test for multiple comparisons for differences at different time points compared to baseline lactate concentration
