# Supplementary material for: Microdialysis and ultrasound elastography for monitoring of localized muscular reaction after pharmacological stimulation in rats
Source: BMC Res Notes. 2018 Sep 3;11:636. doi: 10.1186/s13104-018-3742-6 (PMC6122639; doi:10.1186/s13104-018-3742-6)
Supplement: Supplementary file 3 — Additional file 3: Table S3a. Sizes of investigated areas, groups A, B and C. b. Sizes of investigated areas, groups D and E. c. Sizes of investigated areas, groups F and G. [file 13104_2018_3742_MOESM3_ESM.pdf]

Table S3 a: Sizes of investigated areas, groups A, B and C

	Ringer (A)	Sorbitol (B)	Calcium chloride (C)
**area (mm^2)**	43 [35;44]	39 [35,40]	36 [35;41]
p value	I----- p =0.9817 -----I (A–B)		I----- p >0.9999 ----I (B–C)
	I------------------ p =0.9597 -----------------I (A–C)		

Sizes of evaluated measuring fields for ultrasound strain elastography during continuous application of Ringer solution (A), sorbitol 160 mM (B) and calcium chloride 160 mM (C). Results as median and interquartile range. One-way ANOVA with post hoc Sidak test for multiple comparisons for differences between the groups

Table S3 b: Sizes of investigated areas, groups D and E

	Caffeine (D)	Ringer (E)	p value
**area (mm^2)**	46 [42;46]	42 [40;45]	0.9463

Sizes of evaluated measuring fields for ultrasound strain elastography after bolus application of caffeine 160 mM (D) and Ringer solution (E). Results as median and interquartile range. One-way ANOVA with post hoc Sidak test for multiple comparisons for differences between the groups

Table S3 c: Sizes of investigated areas, groups F and G

	Halothane (F)	Soybean oil (G)	p value
**area (mm^2)**	45 [44;48]	45 [43;46]	>0.9999

Sizes of evaluated measuring fields for ultrasound strain elastography following bolus application of halothane 10 %vol (F) and soybean oil (G). Results as median and interquartile range. One-way ANOVA with post hoc Sidak test for multiple comparisons for differences between the groups
