# Supplementary material for: Microdialysis and ultrasound elastography for monitoring of localized muscular reaction after pharmacological stimulation in rats
Source: BMC Res Notes. 2018 Sep 3;11:636. doi: 10.1186/s13104-018-3742-6 (PMC6122639; doi:10.1186/s13104-018-3742-6)
Supplement: Supplementary file 4 — Additional file 4: Table S4a. Differences in muscle elasticity, groups A, B and C. b. Differences in muscle elasticity, groups D and E. c. Differences in muscle elasticity, groups F and G. [file 13104_2018_3742_MOESM4_ESM.pdf]

Table S4 a: Differences in muscle elasticity, groups A, B and C

	Ringer (A)		Sorbitol (B)		Calcium chloride (C)	
**time** (min)	**Δelasticity**	p value	**Δelasticity**	p value	**Δelasticity**	p value
**1**	**-1** [-5;0]	0.9942	**0** [-2;2]	>0.9999	**-1** [-3;2]	>0.9999
**5**	**-1** [-3;1]	>0.9999	**4** [-3;7]	0.9895	**-1** [-4;1]	0.9987
**10**	**-2** [-7;1]	0.8577	**4** [1;7]	0.5807	**-3** [-5;1]	0.9895
**15**	**-1** [-12;1]	0.7318	**5** [0;12]	0.2051	**-7** [-9;-1]	0.2788
**20**	**-3** [-10;1]	0.4295	**6** [-1;10]	0.4178	**-4** [-9;1]	0.5807
**25**	**-4** [-11;8]	0.9186	**2** [0;10]	0.6364	**-4** [-11;1]	0.5250
**30**	**-2** [-10;7]	0.9730	**4** [1;8]	0.5250	**-1** [-9;3]	0.9819
**40**	**-4** [-9;5]	0.8907	**3** [-1;6]	0.9548	**-3** [-11;2]	0.7430
**50**	**-4** [-9;3]	>0.9999	**4** [1;8]	0.7915	**-5** [-9;1]	0.5807
**60**	**-6** [-10;4]	0.5809	**5** [-1;8]	0.7430	**-5** [-11;0]	0.2788

Differences in mean muscle elasticity determined by ultrasound strain elastography during continuous application of Ringer solution, sorbitol 160 mM and calcium chloride 160 mM. Negative values reveal increased muscle stiffness, positive values decreased stiffness compared to baseline. Data presented as median and interquartile range, 2-way ANOVA with post-hoc Sidak test for multiple comparisons

Table S4 b: Differences in muscle elasticity, groups D and E

	Caffeine (D)		Ringer (E)	
**time** (min)	**Δelasticity**	p value	**Δelasticity**	p value
**1**	**-3** [-4;1]	0.8840	**2** [-4;5]	0.9997
**5**	**-3** [-6;-2]	0.7099	**-1** [-1;6]	0.9978
**10**	**-1** [-2;2]	>0.9999	**-3** [-4;3]	0.9991
**15**	**3** [-1;3]	0.9906	**-5** [-8;4]	0.7261
**20**	**-1** [-3;0]	0.9998	**-3** [-7;3]	0.9042
**25**	**-2** [-3;4]	>0.9999	**-3** [-5;2]	0.9834
**30**	**-2** [-5;2]	0.9975	**-4** [-9;1]	0.3836
**40**	**0** [-5;1]	>0.9999	**-4** [-8;-2]	0.2084
**50**	**1** [-7;2]	0.9906	**-4** [-7;0]	0.5524
**60**	**-4** [-5;1]	0.8840	**-8** [-9;-2]	0.8027

Differences in mean muscle elasticity determined by ultrasound strain elastography following bolus application of caffeine 160 mM (D) and Ringer solution (E). Negative values reveal increased muscle stiffness, positive values decreased stiffness compared to baseline. Data presented as median and interquartile range, 2-way ANOVA with post-hoc Sidak test for multiple comparisons

Table S4 c: Differences in muscle elasticity, groups F and G

	Halothane (F)		Soybean oil (G)	
**time** (min)	**Δelasticity**	p value	**Δelasticity**	p value
**1**	**-4** [-5;0]	0.9159	**2** [-3;6]	0.9998
**5**	**-3** [-4;0]	0.9915	**-1** [-6;8]	>0.9999
**10**	**1** [-3;2]	>0.9999	**0** [-7;4]	>0.9999
**15**	**1** [-2;3]	>0.9999	**1** [-8;6]	>0.9999
**20**	**-1** [-2;2]	>0.9999	**1** [-5;8]	>0.9999
**25**	**2** [-3;3]	>0.9999	**0** [-6;3]	>0.9999
**30**	**2** [-2;3]	>0.9999	**1** [-6;5]	>0.9999
**40**	**0** [-1;1]	>0.9999	**1** [-6;13]	0.5560
**50**	**0** [-4;2]	>0.9999	**-2** [-8;8]	0.9994
**60**	**0** [-4;0]	0.9997	**-2** [-7;11]	0.9761

Differences in mean muscle elasticity determined by ultrasound strain elastography following bolus application of halothane 10 %vol (F) and soybean oil (G). Negative values reveal increased muscle stiffness, positive values decreased stiffness compared to baseline. Data given as median and interquartile range, 2-way ANOVA with post-hoc Sidak test for multiple comparisons
